# Laparoscopic Management of Para-Caecal Hernia With Small Bowel Obstruction: A Case Report

**DOI:** 10.7759/cureus.42642

**Published:** 2023-07-29

**Authors:** Ehsan Ulhaq, Ioannis Loufopoulos, Vijitha Chandima Halahakoon

**Affiliations:** 1 General and Colorectal Surgery, Colchester Hospital/East Suffolk North Essex NHS Foundation Trust, Colchester, GBR

**Keywords:** laparoscopic approach, small bowel obstruction, pericaecal hernia, paracaecal hernia, internal hernia

## Abstract

Para-caecal hernias are a rare type of internal hernias. They can cause bowel obstruction, leading to strangulation of the bowel. As such, urgent diagnosis and appropriate management are important. Both laparoscopic and open approaches are options in terms of surgical treatment. We report a case of a para-caecal hernia causing small bowel obstruction and highlight the laparoscopic approach as a feasible and effective way of management.

## Introduction

Internal hernias are a type of hernia, where a portion of the abdominal viscera, like the intestine, protrudes through an anatomical or non-anatomical defect within the peritoneal cavity. According to the Ghahremani classification [1], internal hernias are classified as para-duodenal, hernias through the foramen of Winslow, and trans-mesenteric, para-caecal, inter-sigmoid, trans-omental and retro-anastomotic hernias. The underlying causes resulting in the development of this type of hernia are multiple, including congenital defects and caves, previous surgical interventions and trauma to the abdomen. If internal hernias are not managed duly and expeditiously, they can result in strangulation and perforation of the bowel, increasing morbidity and mortality.

Para-caecal hernias, also named peri-caecal hernias, is the herniation of a segment of the small bowel through a sack of peritoneum near the cecum. Para-caecal hernias, which constitute 0.1%-6.6% of all internal hernias, can cause intestinal obstruction or even strangulation if left untreated [2].

Presentation of peri-caecal hernias is variable, from either being asymptomatic or presenting with abdominal pain, distention and vomiting, indicative of small bowel obstruction. Due to abdominal pain being localized in the right lower quadrant, acute appendicitis is usually a differential diagnosis in such cases. In this regard, detailed history-taking, including previous abdominal trauma, operations, and thorough clinical examination are suggested.

Computed tomography (CT) scan is the investigation of choice to confirm internal herniation and bowel obstruction [3]. Magnetic resonance imaging (MRI) is reserved in case a CT scan is contraindicated.

Treatment options may include both conservative management and surgical interventions. Supportive/conservative management includes restriction of oral intake, decompression by nasogastric tube, intravenous fluid replacement and pain optimization. If the conservative approach fails with no improvement in symptoms or deteriorating physical signs with a risk of complications like strangulation or bowel ischemia, early surgical intervention is required. Surgical intervention includes the laparoscopic or open approach depending on the patient’s condition, urgency and surgeon’s expertise.

We describe a case of para-caecal herniation of the small bowel as a rare cause of bowel obstruction, which was successfully managed by a laparoscopic approach.

## Case presentation

**Figure 1:**
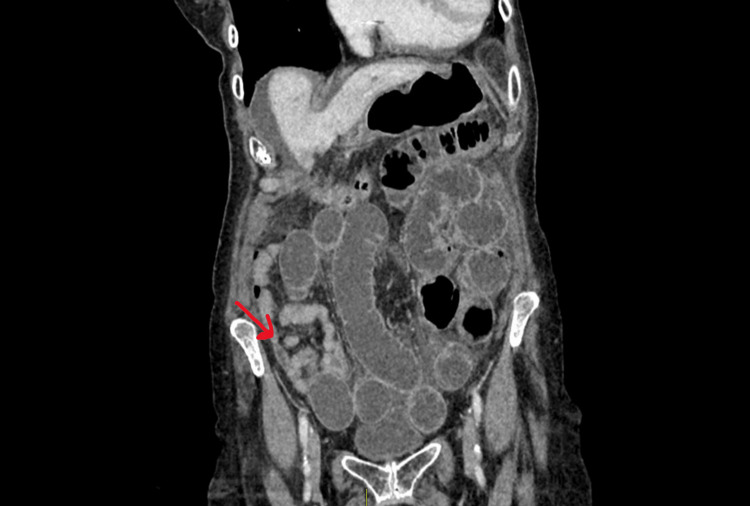
CT showing proximal bowel dilation with distal small bowel collapse with the transition point in the right iliac fossa

**Figure 2:**
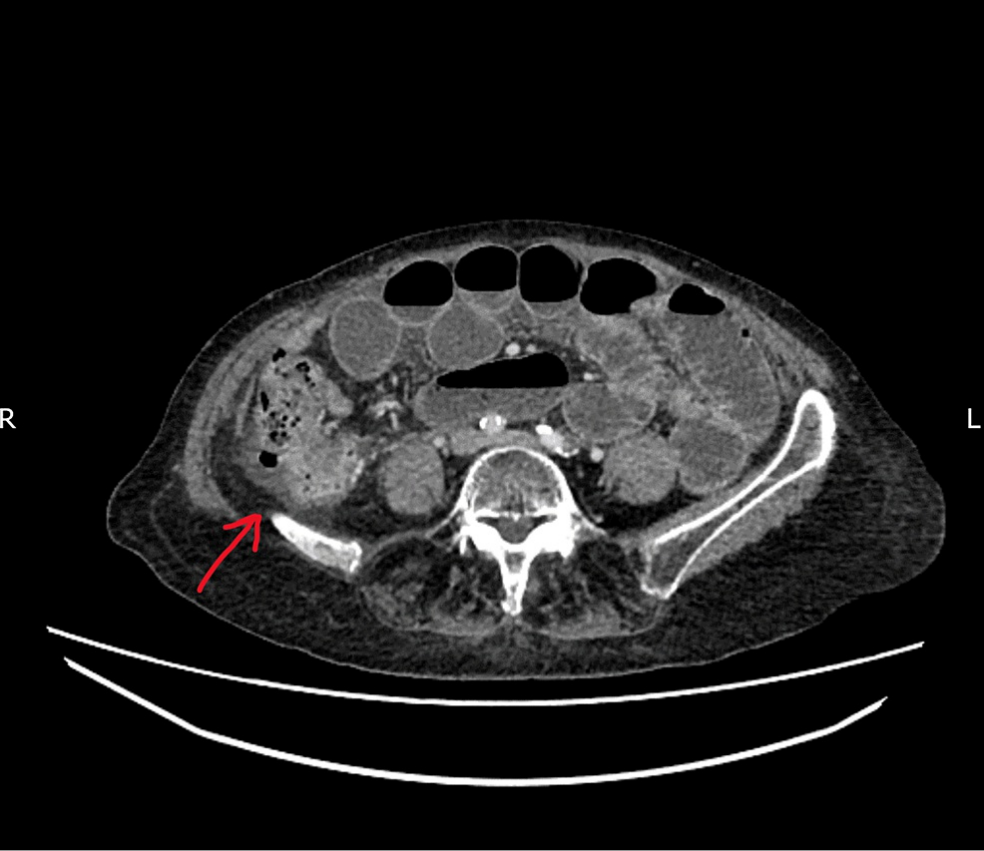
Axial view showing the transition point of the small bowel in the right iliac fossa

An 88-year-old female, who had an appendicectomy in the past, was admitted under the emergency surgical team with a three-day history of central abdominal pain, vomiting and mild abdominal distention. Routine blood tests were normal. CT abdomen and pelvis were performed after intravenous contrast. Scanned in the portovenous phase, CT findings were reported as significant small bowel distention with multiple gas fluid levels with a sudden change in the calibre of the small bowel loop in the right iliac fossa with upstream small bowel distention, suggestive of possible adhesional bowel obstruction (Figures 1-2). Initially, the patient was managed conservatively with nasogastric tube decompression, intravenous fluids, restricted oral intake and gastrograffin challenge, with a presumed diagnosis of adhesional bowel obstruction. Due to the failure of the non-operative management with the persistent abdominal distention and high nasogastric output, the patient was offered emergency laparoscopy.

**Figure 3:**
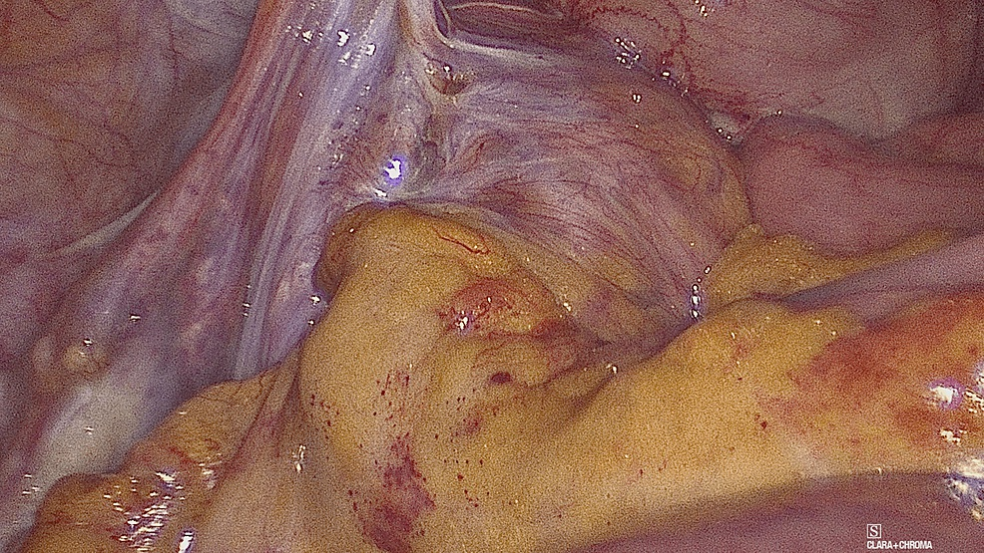
Para-caecal sac in the right iliac fossa containing a loop of small bowel

**Figure 4:**
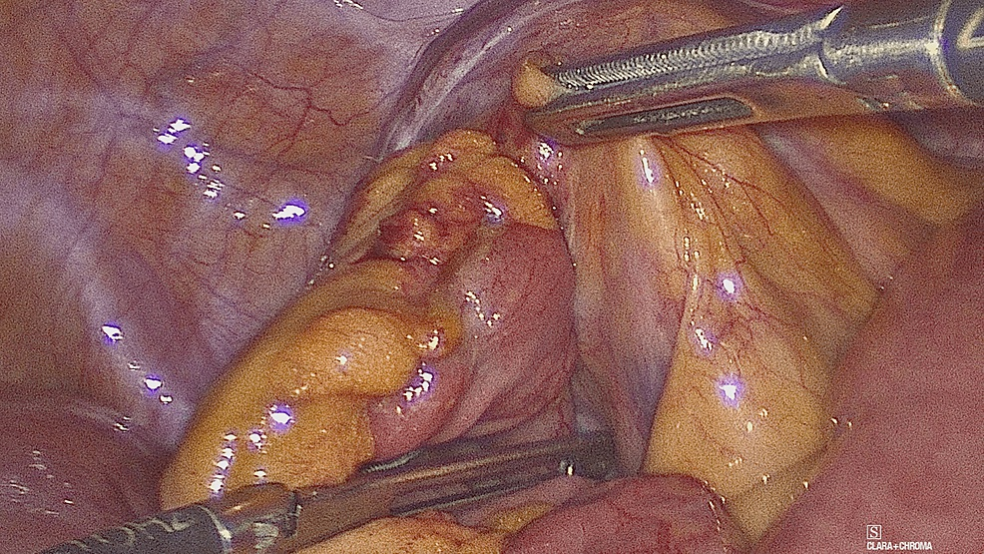
Release of the herniated bowel from the sac

Intra-operative findings (Figures 3-4) revealed para-caecal peritoneal folds forming a para-caecal recess containing a loop of small bowel resulting in intestinal obstruction and dilation of proximal small bowel with the collapsed distal small bowel loops. Due to the wide neck of the hernial sac, there was no strangulation of the bowel. Laparoscopic de-roofing of the cavity was performed to free the small bowel and prevent the recurrence. Post-operative recovery was uneventful, and the patient was discharged on post-operative day four.

## Discussion

Internal hernias are defined as “the protrusion of a viscus, usually small bowel, through a peritoneal or mesenteric aperture within the abdominal cavity” [4]. Although the incidence of internal hernia is less than 1%, they are a potential cause of small bowel obstruction in 5.8% of cases [1,5]. Internal hernias are either congenital or acquired. Among the potential causes, the most significant ones are previous abdominal surgical interventions (e.g. bariatric procedures like gastric bypass), trauma or inflammation of the peritoneum [3]. Due to its mechanical nature, bowel obstruction within an internal hernia can easily progress to strangulation and subsequently to ischemic compromise. This constitutes a surgical emergency, warranting urgent resection of the non-viable bowel. This not only affects patients’ quality of life (stoma formation, prolonged hospitalization) but also poses a risk to their life.

Para-caecal hernias are a rare type of internal hernias (13%) [3] and are scarcely reported in the current literature. They are classified into four subtypes: superior ileocecal recess, retro-caecal recess, inferior ileocaecal recess and para-colic sulcus [2].

Following a literature review from 2016 onwards, we identified 17 cases describing the diagnosis and surgical management of this rare cohort of patients. Not many cases have been described so far, and high clinical suspicion is required from the clinician, as it constitutes a pathology that can cause mechanical bowel obstruction and quickly progress to strangulation.

The clinical presentation varies from being asymptomatic to presenting with sub-acute or acute intestinal obstruction requiring urgent or expedited surgical intervention. In this context, the pre-operative diagnostic approach is a crucial step in its early identification. Although plain abdominal X-rays can suggest bowel obstruction, they have a poor role in the diagnosis of internal herniation. CT scan is the imaging modality of choice for assessing internal hernias with high accuracy [3,6,7] although in our case, it failed to confirm the para-caecal herniation of the small bowel. When in doubt, direct vision under diagnostic laparoscopy or laparotomy is required. Perioperative findings reported in the literature vary from simply obstructed bowel, like in our patient, to strangulated hernias with ischemic bowel requiring resection. In our case, the sub-acute nature of small bowel obstruction and CT interpretation of possible adhesional small bowel obstruction resulted in initial conservative management as a reasonable approach. However, later, the intra-operative findings confirmed the cause of bowel obstruction as an internal herniation of a small bowel loop into a para-caecal recess.

Historically, both open and laparoscopic management have been reported for such hernias. However, the laparoscopic approach as a minimally invasive surgical procedure has become more popular in the management of such cases. In addition to early recovery, shorter hospitalisation and fewer post-operative complications, such as pain and wound infections, the laparoscopic approach has overall reduced morbidity and mortality compared to the open one [2,8]. An open laparotomy is an option when the patient is haemodynamically unstable and early intervention is required or the surgeon does not have the appropriate expertise.

Table 1 lists studies based on para-caecal hernia from 2016 to the present [2,4-7,9-20].

**Table 1:** A literature review of para-caecal hernia from 2016 to the present

Authors	Year	Age (years)	Sex	Previous abdominal operations	Surgical management
Sasaki et al. [9]	2016	65	Male	None	Laparoscopy
Ogami et al. [2]	2016	92	Male	Cholecystectomy	Laparoscopy
Ito et al. [6]	2017	83	Male	None	Laparotomy
Chia et al. [10]	2017	32	Male	None	Laparoscopy
Tayran et al. [11]	2017	75	Female	None	Laparoscopy
Inukai et al. [12]	2018	54	Male	None	Laparoscopy-assisted
Menezes et al. [13]	2018	40	Male	None	Laparotomy
Otani et al. [14]	2018	83	Female	None	Laparoscopy
Aljaberi et al. [15]	2019	16	Male	None	Laparotomy converted from Laparoscopy
Yokota et al. [16]	2019	86	Female	Appendicectomy	Laparotomy
Plua-Muniz et al. [17]	2020	84	Female	None	Laparoscopy
Mansfield et al. [18]	2020	80	Female	Appendicectomy	Laparotomy
Gurumurthi et al. [19]	2021	89	Male	N/A	Laparotomy
Al-Ardah et al. [5]	2021	68	Female	N/A	Laparoscopy
Son et al. [7]	2022	64	Female	None	Laparoscopy
Pentakota et al. [20]	2023	60s	Male	N/A	Laparotomy
Mare et al. [4]	2023	54	Female	Appendicectomy, cholecystectomy, gastric band insertion, band removal and sleeve gastrectomy	Laparoscopy

## Conclusions

We reported a case of laparoscopic management of a para-caecal hernia causing small bowel obstruction. Awareness of various types and clinical presentations of internal hernias can help in the pre-operative diagnosis. Laparoscopy is not only diagnostic but also effective in dealing with such hernias effectively.

## References

[1] Ghahremani GG (1984). Internal abdominal hernias. Surg Clin North Am.

[2] Ogami T, Honjo H, Kusanagi H (2016). Pericecal hernia manifesting as a small bowel obstruction successfully treated with laparoscopic surgery. J Surg Case Rep.

[3] Lanzetta MM, Masserelli A, Addeo G (2019). Internal hernias: a difficult diagnostic challenge. Review of CT signs and clinical findings. Acta Biomed.

[4] Mare H, Tjhin W (2023). Successful laparoscopic management of pericaecal hernia causing small bowel obstruction. Cureus.

[5] Al-Ardah M, Sisodia H, Rottenburg H, Clarke M (2021). Laparoscopic management of strangulated paracaecal hernia causing small bowel obstruction. Case report and review of the literature. J Surg Case Rep.

[6] Ito S, Takeda R, Kokubo R (2017). Retrocecal hernia preoperatively diagnosed by computed tomography: a case report. Int J Surg Case Rep.

[7] Son TQ, Hoc TH, Thanh Tung T, Long VD, Dat NT, Dinh NQ, Huong TT (2022). Laparoscopic surgery for intestinal obstruction caused by an internal paracecal hernia. Case Rep Gastroenterol.

[8] O'Connor DB, Winter DC (2012). The role of laparoscopy in the management of acute small-bowel obstruction: a review of over 2,000 cases. Surg Endosc.

[9] Sasaki K, Kawasaki H, Abe H, Nagai H, Yoshimi F (2016). Retrocecal hernia successfully treated with laparoscopic surgery: a case report and literature review of 15 cases in Japan. Int J Surg Case Rep.

[10] Chia DK, Tay KV, Kow A, So J, Shabbir A, Kim G (2019). Paracaecal hernia: uncommon but important cause of small bowel obstruction successfully managed with laparoscopic surgery. ANZ J Surg.

[11] Tayaran A, Abdulrasool H, Bui HT (2017). Paracaecal hernia: a case report on the evolving role of laparoscopy. Int J Surg Case Rep.

[12] Inukai K, Tsuji E, Uehara S (2018). Paracecal hernia with intestinal ischemia treated with laparoscopic assisted surgery. Int J Surg Case Rep.

[13] Menezes R, Kamble R, Joshi A, Chaudhari K (2018). Closed loop small bowel obstruction due to paracaecal internal herniation: a lesson in rarity. BMJ Case Rep.

[14] Otani H, Makihara S (2018). Laparoscopic surgery for intestinal obstruction caused by an internal paracecal hernia. Acta Med Okayama.

[15] AlJaberi LM, Salameh AK, Mashalah RM, AbuMaria A (2019). Pericecal hernia in a pediatric patient: case report and literature review. Int J Surg Case Rep.

[16] Yokota T, Otani K, Yoshida J (2019). Paracecal hernia due to membranous adhesion of the omentum to the right paracolic gutter. Surg Case Rep.

[17] Plua-Muñiz K, Sanchez-Gonzalez J, Bailon-Cuadrado M, Pacheco-Sanchez D (2020). Small bowel obstruction caused by pericaecal hernia resolved with a laparoscopic approach. Ann R Coll Surg Engl.

[18] Mansfield M, Wright N (2020). Pericaecal hernia: an unusual cause of bowel obstruction. ANZ J Surg.

[19] Gurumurthi B, Luffman W, Rehman M, Fatayer T, Sharma A (2021). Uncommon presentation of small bowel pericaecal hernia in an octogenarian. ANZ J Surg.

[20] Pentakota N, Abuji K, Vaddavalli VV, Sakaray Y (2023). Retrocecal hernia with small bowel obstruction: a review of literature. BMJ Case Rep.

